# Antimicrobial Resistance, FlaA Sequencing, and Phylogenetic Analysis of *Campylobacter* Isolates from Broiler Chicken Flocks in Greece

**DOI:** 10.3390/vetsci8050068

**Published:** 2021-04-21

**Authors:** George Natsos, Niki K. Mouttotou, Emmanouil Magiorkinis, Anastasios Ioannidis, Maria Magana, Stylianos Chatzipanagiotou, Konstantinos C. Koutoulis

**Affiliations:** 1 Department of Poultry Diseases, Veterinary Faculty, University of Thessaly, 43100 Karditsa, Greece; natsos@vet.uth.gr; 2 Ministry of Rural Development and Foods, National Reference Laboratory for *Salmonella* and Antimicrobial Resistance, 34100 Chalkida, Greece; nmouttotou@minagric.gr; 3 Department of Laboratory Haematology, General Hospital for Chest Diseases “Sotiria”, 11527 Athens, Greece; mayiork@med.uoa.gr; 4 Department of Nursing, Faculty of Human Movement and Quality of Life Sciences, University of Peloponnese, 23100 Sparta, Greece; tasobi@uop.gr (A.I.); mariamgn91@gmail.com (M.M.); 5 Department of Medical Biopathology, Medical School–Eginition Hospital, National and Kapodistrian University of Athens, 15772 Athens, Greece; schatzi@med.uoa.gr

**Keywords:** *Campylobacter* spp., poultry, antimicrobial resistance, flaA typing, phylogenetic trees, Greece

## Abstract

Human campylobacteriosis caused by thermophilic *Campylobacter* species is the most commonly reported foodborne zoonosis. Consumption of contaminated poultry meat is regarded as the main source of human infection. This study was undertaken to determine the antimicrobial susceptibility and the molecular epidemiology of 205 *Campylobacter* isolates derived from Greek flocks slaughtered in three different slaughterhouses over a 14-month period. A total of 98.5% of the isolates were resistant to at least one antimicrobial agent. In terms of multidrug resistance, 11.7% of isolates were resistant to three or more groups of antimicrobials. Extremely high resistance to fluoroquinolones (89%), very high resistance to tetracycline (69%), and low resistance to macrolides (7%) were detected. FlaA sequencing was performed for the subtyping of 64 *C. jejuni* and 58 *C. coli* isolates. No prevalence of a specific flaA type was observed, indicating the genetic diversity of the isolates, while some flaA types were found to share similar antimicrobial resistance patterns. Phylogenetic trees were constructed using the neighbor-joining method. Seven clusters of the *C. jejuni* phylogenetic tree and three clusters of the *C. coli* tree were considered significant with bootstrap values >75%. Some isolates clustered together were originated from the same or adjacent farms, indicating transmission via personnel or shared equipment. These results are important and help further the understanding of the molecular epidemiology and antimicrobial resistance of *Campylobacter* spp. derived from poultry in Greece.

## 1. Introduction

*Campylobacter* spp. are ubiquitous bacteria, able to colonize mucosal surfaces, usually the intestinal tract, of most mammalian and avian species [1,2]. Thermophilic *Campylobacter* spp. are essentially commensal in birds and insignificant for poultry health [3]. However, they are of high importance to food safety and public health, since they are recognized as the leading cause of bacterial foodborne diarrheal disease worldwide [1,4]. Birds carrying *Campylobacter* are asymptomatic colonizers without any clinical signs [5]. Broilers are considered *Campylobacter* free after hatching, since most evidence suggests that vertical transmission plays a minor role, if any [1], and, in general, broiler flocks remain *Campylobacter* free for the first two weeks (the so-called lag phase) [6]. This lag phase is likely to be an inherent property of the chick. An inhibitory effect produced by commensal organisms in the gut of young chicks [7], the presence of maternal antibodies, which may be protective and which decline by about 14 days of age [8], and antimicrobial treatment contribute to the existence of the lag phase. As chickens are coprophagic, fecal shedding is presumably an important factor in the dissemination of organisms around large broiler flocks once the first bird becomes colonized. Certainly, once flock colonization is detected, bird-to-bird transmission within flocks is extremely rapid, and the majority (up to 100%) of birds in a positive flock are colonized within only a few days [6,9].

Consumption of poultry meat or ready-to-eat meat cross-contaminated by contact with raw poultry products constitutes the main risk factor for sporadic human infection [1,10,11]. Thus, control of campylobacteriosis is commonly focused on reducing the occurrence of *Campylobacter* in broiler meat [12]. *C. jejuni* is the predominant species isolated from poultry samples, followed by *C. coli*, with other *Campylobacter* species such as *C. lari* being less detected [13]. However, the predominance of *C. coli* has been reported in Greece [14,15] and other southern European countries [13], which could be attributed to the differences in climatic conditions, environmental reservoirs, housing systems of broiler chickens, and age of slaughter between northern and southern Europe [16]. *C. jejuni* is, as well, considered responsible for the majority of human campylobacteriosis, followed by *C. coli*, and, rarely, by other emerging *Campylobacter* species, including *C. concisus*, *C. ureolyticus*, *C. upsaliensis*, and *C. lari* [4]. 

In the European Union (EU), campylobacteriosis has been the most commonly reported cause of human foodborne zoonoses since 2005 [17,18]. Antimicrobial treatment is usually not required, but effective treatment may shorten the duration of illness [19]. In cases where antimicrobial treatment is needed, macrolides (mostly erythromycin and azithromycin) and fluoroquinolones (e.g., ciprofloxacin) are considered as the first and second choices of antimicrobials, respectively [20,21]. Since a rapidly increasing proportion of *Campylobacter* strains worldwide have been found to be resistant to these antimicrobials, attention should be paid to choosing the most appropriate antimicrobial treatment [19]. Infection with antimicrobial-resistant *Campylobacter* may lead to suboptimal outcomes of antimicrobial treatments or even treatment failure [22]. Therefore, other antimicrobials such as gentamicin, carbapenems, and amoxicillin-clavulanic acid could be alternatively used for the treatment of systemic *Campylobacter* infections [23]. Transmission of antimicrobial resistance from food animals to humans can occur via the food chain. Therefore, food animals are a significant reservoir of antimicrobial-resistant zoonotic pathogens [24]. Consequently, the estimation of antimicrobial susceptibility of *Campylobacter* strains derived from animal samples is crucial. The World Health Organization, therefore, has published a list of critically important antimicrobials for human medicine, emphasizing the importance of prudent use of antimicrobials both in human and veterinary medicine [25].

Due to the impact of *Campylobacter* on public health, epidemiological investigations analyzing the clonality of the isolated strains are very important, in order to trace the sources and routes of transmission, to follow up the temporal and geographic distribution of important phenotypic characteristics, and to develop effective strategies for the control and prevention of the pathogen spread, especially inside the food chain [26,27]. The subtyping of clinical, animal, and food isolates remains an important requirement for epidemiological studies in order to (1) trace sources and routes of transmission of human infections; (2) identify and monitor, temporally and geographically, specific strains with important phenotypic characteristics; and (3) develop strategies to control organisms within the food chain [28]. Classical pulsed-field gel electrophoresis (PFGE) and amplified fragment length polymorphism (AFLP), as well as flaA typing based on the restriction analysis of PCR-amplified fragments or sequencing of the flagellin-encoding gene, have been described for *Campylobacter* [29,30,31]. Although, multilocus sequence typing (MLST) has been described as the gold standard method in this field, MLST is still time consuming and expensive and, therefore, not feasible for routine testing [30,31]. 

The aims of the present study were multiple: (1) to determine the antimicrobial resistance of *Campylobacter* isolates derived from Greek flocks in relation to common antimicrobial substances used in poultry practice and for human medicine, (2) to subtype them using the flaA gene sequencing typing technique, and (3) to perform a phylogenetic analysis in order to study their molecular epidemiology.

## 2. Materials and Methods

### 2.1. Experimental Design

The experimental procedure was conducted in commercial flocks. Therefore, an ethical approval from the University’s Animal Ethics Committee was not required. Samples were collected from 142 slaughter batches, originating from 60 different poultry farms between February 2014 and March 2015 [15]. Caeca were randomly selected from 10 birds per batch during evisceration and pooled into a sterile bag. Neck skin samples of five birds from the processing line after chilling were also taken, using a clean pair of latex gloves and put into a sterile bag. After the sampling, the acquired samples were sent, in an insulated box containing ice packs to maintain a low temperature, within a few hours of the same day to Veterinary Laboratory of Chalkida, where bacteriological analyses were performed.

### 2.2. Sample Analysis

*Campylobacter* spp. recovered from the caecal contents using the technique of direct isolation, in which 10 μL of each caecal sample, previously homogenized by adding Peptone Salt solution (Merck, Darmstadt, Germany), were plated on the selective medium, modified Charcoal Cefoperazone Deoxycholate Agar (mCCDA) (Oxoid, Dardilly, France), followed by incubation for 44 ± 4 h at 41.5 ± 1 °C under microaerobic conditions (5% O_2_, 10% CO_2_, and 85% N_2_). For each positive plate, if necessary, up to five typical *Campylobacter* colonies were then subcultured onto plates of Columbia Blood Agar (Oxoid, Dardilly, France) for further characterization, in accordance with standard procedure of International Organization for Standardization (ISO) 10272-1 [32]. The flock was considered *Campylobacter*-positive, when at least one confirmed *Campylobacter* isolated from a colony yielded a positive result by PCR procedure.

For the recovery of *Campylobacter* from the skin of carcasses, the procedure described in ISO 10272 was followed. For the detection of *Campylobacter*, 10 g of neck skin was placed in a sterile bag and diluted 1:10 with selective pre-enrichment Bolton Broth solution (Oxoid, Dardilly, France). The mix was then homogenized for 1 min in a peristaltic homogenizer and the final suspension was incubated under microaerobic conditions for 4 h at 37 °C and then for 44 ± 4 h at 41.5 ± 1 °C. Subsequently, 10 μL of the suspension were plated onto mCCDA and Butzler (Oxoid, Dardilly, France) plates and followed by incubation for 44 ± 4 h at 41.5 ± 1 °C. For each positive plate, up to five colonies typical of *Campylobacter* were subcultured onto Columbia Blood Agar plates for further characterization, according to standard method of ISO 10272-1:2006. 

### 2.3. Antimicrobial Susceptibility Testing

For each *Campylobacter*-positive sample, antimicrobial susceptibility testing to ciprofloxacin, nalidixic acid, erythromycin, streptomycin, gentamicin, and tetracycline was performed. Antimicrobial disks for the disk diffusion method were obtained from Oxoid, Dardilly, France. Disk diffusion method in Mueller–Hinton agar enriched with 5% defibrinated sheep’s blood was performed. Sterile cotton-tipped swabs were used to inoculate broth culture diluted to match a 0.5 McFarland turbidity standard onto Mueller–Hinton blood agar plates to produce a confluent lawn of bacterial growth. After the inoculum on the plates was dried, antimicrobial disks were distributed over the inoculated plates using an Antimicrobial Susceptibility testing Disk Dispencer (Oxoid, Dardilly, France). These plates were then incubated at 42 °C for 24 h under microaerobic conditions (5% O_2_, 10% CO_2_, and 85% N_2_). Isolates with insufficient growth after 24 h of incubation were re-incubated immediately and inhibition zone was read after a total of 40–48 h of incubation. *Campylobacter jejuni* ATCC 33560 was used as a quality-control (QC) strain and the acceptable ranges of Clinical and Laboratory Standards Institute (CLSI M45) were followed. Since there were no antimicrobial susceptibility breakpoints for disk diffusion method specific with respect to *Campylobacter* for nalidixic acid, gentamicin, and streptomycin provided by CLSI M45, breakpoints of *Enterobacteriaceae* were used (CLSI M100). The concentrations of antimicrobial agents tested in this study along with the zone diameter breakpoints are shown in Table 1.

### 2.4. FlaA Sequencing

A PCR procedure was performed on the DNA extracts of 122 *Campylobacter* isolates. The primers used (FLA4F and FLA630R) were composed by Eurofins Genomics, were in freeze-drying state, and were selected based on a study of Meinersmann et al. [33]. Sanger sequencing was performed in a 3130 Genetic Analyzer (Applied Biosystems Life Technologies Ltd., Paisley, UK). For the sequencing of the flaA gene, DNA STAR’s Laser gene Evolution Suite software was used. All sequences were submitted to GenBank and issued accession numbers (MW713238–MW713296 for *C. coli* sequences and MW713297–MW713360 for *C. jejuni* sequences).

### 2.5. Phylogenetic Trees

All available flaA sequences for *C. jejuni* and *C. coli* were downloaded from different geographic regions. For *C. jejuni*, the analysis involved 64 sequences isolated in our study plus 960 flaA reference sequences (RS) downloaded from the GenBank database. For *C. coli,* the numbers were 58 sequences plus 74 flaA reference sequences, respectively. Phylogenetic analysis was performed, estimating the genetic distances between sequences using Tamura–Nei model [34]. Phylogenetic trees were constructed using the neighbor-joining method and the reliability of phylogenetic clusters was assessed using bootstrapping analysis of 1000 copies. The trees were drawn to scale, with branch lengths in the same units as those of the evolutionary distances used to infer the phylogenetic trees. The alignment of all sequences was performed by Cluster W algorithm using the MEGA 5 version 5.0 software, while all positions containing gaps and missing data were manually edited.

## 3. Results

### 3.1. Antimicrobial Resistance 

According to CLSI antimicrobial susceptibility breakpoints, 86.7% of *Campylobacter* isolates from caecal samples were classified as resistant to ciprofloxacin, 87.6% as resistant to nalidixic acid, and 77.1% as resistant to tetracycline. On the other hand, very low resistance to erythromycin (7.6%) and streptomycin (11.4%) and no resistance to gentamicin were found. Similar results came from antimicrobial resistance testing of neck skin samples (Table 2). The results of antimicrobial susceptibility in relation to the species of *Campylobacter* isolates are shown in Table 3.

Only three strains were susceptible to all antimicrobial agents. Additionally, 13 out of 205 (6.3%) *Campylobacter* isolates showed co-resistance to ciprofloxacin and erythromycin, whereas 24 out of 205 (11.7%) were resistant to three or more groups of antimicrobials (i.e., fluoroquinolones, macrolides, tetracyclines, aminoglycosides).

### 3.2. FlaA Sequencing

A high degree of genetic diversity was revealed, with a total of 38 different nucleotide types that corresponded to 15 different peptide types. Peptide type 1 was the most predominant since it was recovered from 58 *Campylobacter* isolates. Of the isolates, 92.6% (113 out of 122) showed exact match with the already registered ones in the international database, whereas 7.4% (9 out of 122) displayed partial match; namely, the isolates had a rate of homology though preserving different regions inside the sequences. Some isolates shared the same nucleotide and peptide type in an exact match with the registered types in the international database, suggesting the occurrence of clonality. Moreover, some of these isolates shared common antimicrobial profile (e.g., peptide type 1-DNA type 66).

### 3.3. Phylogenetic Trees

**Figure 1:**
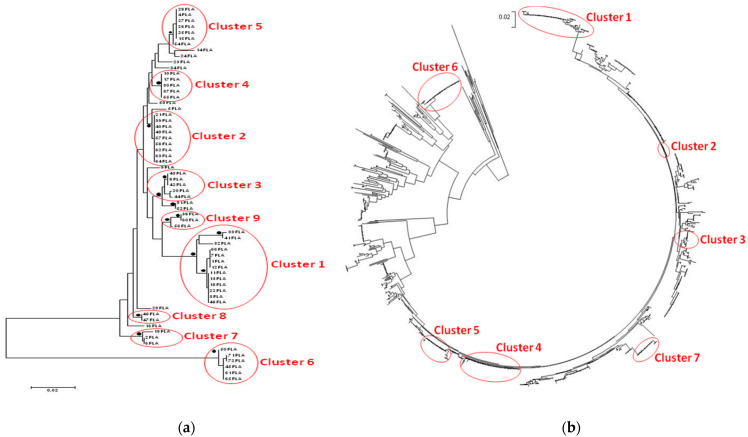
*Campylobacter jejuni* phylogenetic trees. Bullets represent clades, which had bootstrap values >75% of permuted trees. (**a**) The analysis involved 64 sequences. Most of the sequences were organized in nine significant clusters supported with high bootstrap values. (**b**) The optimal tree with the sum of branch length = 4.34019783 is shown.

**Figure 2:**
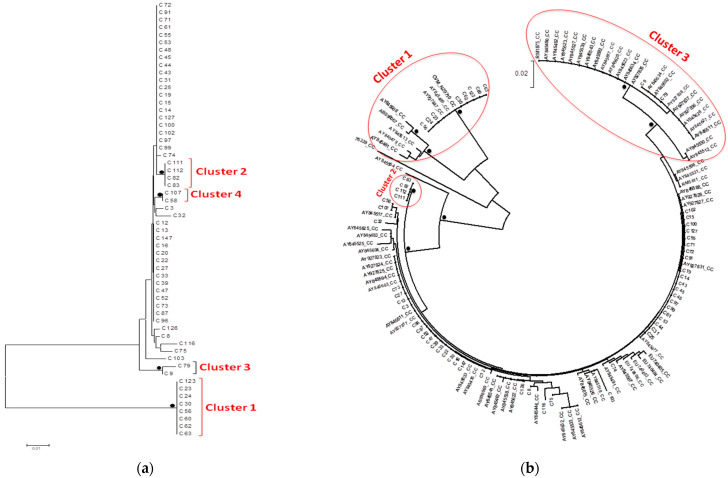
*Campylobacter coli* phylogenetic trees. Bullets represent clades, which had bootstrap values >75% of permuted trees. (**a**) The optimal tree with the sum of branch length = 0.21165541 is shown. The analysis involved 58 sequences. Most of the sequences were dispersed within the tree, whereas four significant sequence clusters were noticed. (**b**) The optimal tree with the sum of branch length = 0.54 is shown.

The phylogenetic trees of *C. jejuni* and *C. coli* isolates are shown in Figure 1a and Figure 2a. Whereas most of the sequences found to be scattered inside the trees, seven clusters of the *C. jejuni* phylogenetic tree (Figure 1b) and three clusters of the *C. coli* tree (Figure 2b) were considered significant with bootstrap values >75%.

Among the 13 *C. jejuni* isolates of the first cluster, eight shared the same DNA and peptide fla type, while two isolates (03FLA-33FLA) originated from the same poultry farm had the same antimicrobial profile. All eight isolates from the second cluster shared the same DNA and peptide fla type, while there were two pairs (21FLA-63FLA and 40FLA-57FLA) that originated from the same farms and had similar antimicrobial profile. Likewise, in the third cluster, there were two isolates (20FLA-43FLA) that originated from the same farm and shared both identical DNA and peptide fla type and antimicrobial profile. All five isolates from the fourth cluster had the same DNA and peptide fla type and quite similar antimicrobial resistance. In the fifth cluster, there were three isolates (15FLA-27FLA-28FLA) that originated from two adjacent houses of the same farm and shared the same DNA and peptide fla type and antimicrobial profile. All seven clusters included reference sequences isolated from different regions (mainly USA, Europe, Tanzania, and Australia). However, no clear connection between them and the isolates of the current study could be made.

In the first cluster of *C. coli* phylogenetic tree, three of eight isolates (C60-C62-C63) originated from neighboring farms located in the same region and exhibited similar antimicrobial resistance patterns. The second cluster included only four isolates (C82-C83-C111-C112), all of which originated from the same poultry farm and shared similar antimicrobial profiles. Almost all reference sequences in the first and third clusters originated from the USA, with the exception of one sequence that originated from Japan.

## 4. Discussion

The results of our study regarding the antimicrobial resistance are consistent with other studies [20,35]. More specifically, high resistance to ciprofloxacin (89.3%) and nalidixic acid (88.3%) was observed. Similar results were submitted on the view of the obligatory monitoring and report of antimicrobial resistance by Greece in 2014, while the overall resistance to quinolones at the EU level was slightly lower [36]. Resistance to fluoroquinolones in *Campylobacter* spp. was firstly reported in the late 1980s and, since then, there is a continuous increase of resistance to fluoroquinolones [37]. It has been observed that resistance appeared simultaneously with the introduction of these agents in animal production and veterinary medicine [11,22]. Since campylobacteriosis is considered to be a zoonosis, the presence of resistant strains in the food chain also has an influence on human infections [11]. Moreover, it has been noted that the proportion of ciprofloxacin-resistant members of the genus *Campylobacter* in poultry meat is often strikingly similar to the proportion observed in human clinical cases [36]. However, the transmission of fluoroquinolone-resistant bacteria from food-producing animals to humans is difficult to prove, and a recent global report on surveillance of antimicrobial resistance emphasized the need to collect more data of the effects of antimicrobial resistance in foodborne bacteria and human health [38,39]. Besides their excessive use in agriculture, the use of fluoroquinolones for infections other than gastroenteritis, as well as “self-medication”, are often causes of the observed resistance in developing countries [40]. Therefore, traveling to developing countries has been implied to be a risk factor for gaining an infection caused by a resistant *Campylobacter* strain. In the developed world, one reason behind fluoroquinolone resistance might also be their inappropriate empirical use in the treatment of human infections. Patients treated with fluoroquinolones were later found to carry bacteria resistant to these antimicrobial agents [41].

A low percentage (6.8%) of *Campylobacter* among the strains recovered from caeca and neck skin samples was resistant to erythromycin. This result agrees with the respective ones of the EU survey [19]. However, the majority of these isolates revealed multi-antimicrobial-resistance properties, a finding demonstrated in other studies, as well [42]. Resistance to erythromycin, as a rule, corresponds to cross resistance to other macrolides (for example, azithromycin and clarithromycin), as well as to related drugs of the group of lincosamides (in particular, to clindamycin) and streptogramins [43]. Resistance of *Campylobacter* spp. to macrolides has remained in low and stable levels for a long time. However, there is also evidence from some parts of the world that resistance rates to erythromycin and other macrolides in *Campylobacter* species are slowly increasing [44,45]. Since fluoroquinolone resistance is common, the macrolides have become important in the treatment of campylobacteriosis, resulting in the development of macrolide resistance [36]. Use of macrolides in animal production as therapeutic or growth-promoting agents has been considered to be a significant factor in the selection of erythromycin-resistant *Campylobacter* strains [46]. However, acquisition of erythromycin resistance in *Campylobacter* species is a stepwise process and requires prolonged exposure, in contrast to the rapidly evolving fluoroquinolone resistance [47]. Moreover, Hao et al. have shown that erythromycin-resistant *Campylobacter* strains display a fitness disadvantage when compared with susceptible *Campylobacter* strains, which may lead to a low frequency of macrolide resistance in clinical isolates [48].

Regarding the remaining antimicrobial agents, resistance of *Campylobacter* isolates to tetracycline was found to be remarkably high, especially in strains derived from caecal content. Similarly high resistance rates were observed in the recent report of EFSA and ECDC [19]. Tetracyclines can be used in the treatment of campylobacteriosis, except for children under 9 years of age [49]. However, tetracycline resistance has emerged also among *Campylobacter* species [36]. In *Campylobacter* spp. the most common tetracycline resistance mechanism is a plasmid-mediated ribosomal protecting protein, Tet(O), encoded by the *tet*(O) gene [50]. No resistance to gentamicin and low resistance to streptomycin were found. Quite similar results have been observed in most EU members states [19]. Guyard-Nicodème et al. [51] tested the susceptibility of *C. jejuni* strains derived from broiler meat products collected in retail outlets and found similar results with our study for tetracycline and gentamicin. The main mechanism of aminoglycoside resistance in *Campylobacter* spp. is via aminoglycoside-modifying enzymes, which are usually plasmid-borne [20]. Only three *Campylobacter* isolates showed complete susceptibility to all antimicrobial agents tested. Similar results were submitted by Greece in the frame of the EU survey [36]. On the other hand, 7.6% of *C. jejuni* and 5% of *C. coli* were co-resistant to ciprofloxacin and erythromycin. This fact is worrying since these antimicrobial classes constitute the cornerstone in treatment of severe human campylobacteriosis. Moreover, 13.3% of *C. jejuni* and 10% of *C. coli* strains showed multidrug resistance (MDR), defined as resistance or no-susceptibility to at least three antimicrobial classes—fluoroquinolones, macrolides, tetracyclines, or aminoglycosides [52]. The increase of multidrug-resistant *Campylobacter* strains has increased [53,54], posing a serious risk of treatment failures, since there are very few treatment alternatives of campylobacteriosis caused by multidrug-resistant strains [21]. This increase may reflect the overuse of different antimicrobial agents in veterinary medicine and, especially, in poultry production [11,39], as well as in human medicine, especially when administered without medical prescription [40].

In order to determine the antimicrobial resistance of *Campylobacter*, the disk diffusion method was used. Although the agar dilution method used to determine the minimal inhibitory concentration (MIC) is considered the standard antimicrobial susceptibility testing method for thermophilic *Campylobacter* species [31], it is a labor-intensive, time-consuming, and costly test [55]. On the other hand, the disk diffusion method is simple, inexpensive, and can provide reproducible results if it is conducted carefully with appropriate standardization and quality controls [56,57]. The latter method has been standardized by the CLSI. However, according to those standards, it should be used only as a screening method for resistance to erythromycin and ciprofloxacin; a disk diffusion zone of 6 mm (growth up to the edge of a 6-mm disk) indicates resistance, while any inhibition zone would require an MIC determination of susceptibility (CLSI M45). Due to the lack of breakpoints for the rest of the antibiotics, it was decided to use breakpoints of *Enterobacteriacea* provided by CLSI M100 [58]. This study revealed a high-level correlation between the standardized agar dilution method and the agar disk diffusion method for aminoglycosides, quinolone/fluoroquinolones, erythromycin, and tetracycline in evaluating the resistance of *Campylobacter* spp. Several comparisons of agreement between the disk diffusion method and other susceptibility testing methods for *Campylobacter* have been conducted over the years [57,58,59,60,61], some of which have concluded that disk diffusion method could be used as a reliable alternative method for the testing of susceptibility of *Campylobacter* spp. to ciprofloxacin and erythromycin [57,58]. On the other hand, the results of other studies are different and indicate the unreliability of this method and the need of further standardization [60,61].

The selection of antimicrobials was done according to the published data concerning the widely used antimicrobial agents, both in poultry production and in the treatment of human campylobacteriosis, and followed the panel of antimicrobials from the EU protocol for harmonized monitoring of antimicrobial resistance in human *Salmonella* and *Campylobacter* isolates [62]. *Campylobacter* isolates from each positive sample were tested for resistance to ciprofloxacin, nalidixic acid, erythromycin, streptomycin, gentamicin, and tetracycline, as in the recent EU summary report [36].

Phylogenetic analysis of our strains using reference sequences highlighted seven clusters of *C. jejuni* isolates and three clusters of *C. coli* isolates in our study population. Almost all significant clusters included both sequences of the current cross-sectional study and reference sequences. No clear connection between our *C. jejuni* isolates and the reference sequences was found, even though most of the reference sequences originated from the USA. However, almost all reference sequences in the first and third clusters of *C. coli* originated from two surveys conducted in the USA. The first one dealt with isolates from retail chicken products and humans with gastroenteritis in central Michigan [63], while the second one dealt with isolates from the European CampyNet collection and National Antimicrobial Resistance Monitoring System, derived mostly from humans, chicken, cattle, and swine [64]. No safe conclusion could be drawn, though.

Some of the strains grouped in the same cluster and shared similar antimicrobial profile and fla types were derived from the same farms in different sampling time or from adjacent houses of the same farm. This finding indicates persistence of the infective strains in the house during turnaround time and further contamination of subsequent batches and/or infection of equipment and working clothes, leading to the spread of these strains from one house to another. Indeed, *Campylobacter* can be carried via boots and clothes of farm personnel and shared equipment between broiler houses of the same farm [65,66,67]. Moreover, the presence of colonized flocks has been found to be linked to the turnaround time in a broiler house. Periods of over 14 days can decrease the possibility of residual bacterial contamination [65], while the rapid flock turnover contributes to *Campylobacter* carryover with increased risk being reported if houses are restocked within nine days of depopulation [68]. In any case, the biosecurity and hygiene level should be maintained optimally during the empty time, as it is well known that an external reservoir can host multiple *Campylobacter* strains during the empty period, which will allow colonization of the new flock [66].

The presence of isolates with the same fla types and shared antimicrobial resistance patterns collected from different farms within a close distance in the same region could be attributed to vehicles that visit different farms in the same day without applying adequate disinfection, such as feed delivery trucks, vehicles for collection of litter and dead birds, or transport from the hatchery and to processing plants, which act as mechanical vectors and allow the transmission of these strains from each farm to another. Farm personnel and equipment (e.g., feed trucks) can carry *Campylobacter* between broiler houses and onto subsequent or neighboring farms [65]. Although feed is not seen as a high-risk *Campylobacter* contaminant within the broiler house, since the low water activity of the dry feed does not permit *Campylobacter* survival [67], it can be a vehicle for horizontal transmission into the broiler house [11]. Hald et al. [69] showed that the incidence of *Campylobacter* was lower in farms that feed homegrown wheat compared to farms that are dependent on external supplies. Jonsson et al. [70] found that livestock and broiler farms with flocks positive for *Campylobacter* spp. within a few kilometers’ distance constitute significant risks for colonization in broiler flocks. Furthermore, live bird crates being contaminated with *Campylobacter* from previous (or other) flocks are reintroduced on the farm during catching, and quite often these crates undergo inadequate washing at the slaughterhouse [65]. Crates can carry identical genotypes of microorganisms that originated from broiler flock and abattoirs, which suggests that transport crates are responsible for contamination during transport to slaughter or they could contribute to the *Campylobacter* colonization of broiler houses [71]. 

## 5. Conclusions

In conclusion, the cross-sectional study carried out in Greece produced valuable results concerning the antimicrobial resistance and the molecular epidemiology of *Campylobacter* spp. in poultry production countrywide. High resistance to fluoroquinolones and tetracycline and low resistance to macrolides and aminoglycosides was found. A high genetic diversity was found, while some specific flaA types were found to share similar antimicrobial-resistance patterns. Phylogenetic analysis of the isolates revealed eight clusters of *C. jejuni* and three clusters of *C. coli*. Some isolates clustered together originated from the same or adjacent farms, indicating transmission via personnel or shared equipment. No clear connection between the reference sequences used and the isolates of the current study was found. These results are of high importance and constitute the foundation in understanding the molecular epidemiology and susceptibility patterns of *Campylobacter* spp. derived from poultry in Greece.

## Figures and Tables

**Table 1:** Breakpoints of the disk diffusion method used to determine antimicrobial susceptibility of *Campylobacter* isolates.

Antimicrobial Agent	Disk Concentration (μg)	Zone Diameter Breakpoint (mm) ^1^		
		S	I	R
Ciprofloxacin	5	≥24	21–23	≤20
Erythromycin	15	≥16	13–15	≤12
Tetracycline	30	≥26	23–-25	≤22
Nalidixic acid	30	≥19	14–18	≤13
Gentamicin	10	≥15	13–14	≤12
Streptomycin	10	≥15	12–14	≤11

^1^ Zone diameter breakpoints of ciprofloxacin, erythromycin, and tetracycline for *Campylobacter* spp. were recommended by the CLSI M45, whereas those of nalidixic acid, gentamicin, and streptomycin for Enterobacteriaceae were recommended by the CLSI M100. S, susceptible; I, intermediate; R, resistant.

**Table 2:** Antimicrobial susceptibility patterns of *Campylobacter* spp., identified by the disk diffusion method, according to the sample tested ^1^.

Antimicrobial Agent	Caecal Samples				Neck Skin Samples			
	No. of *Campylobacter* Isolates ^2^			% of Resistant Isolates	No. of *Campylobacter* Isolates ^2^			% of Resistant Isolates
	S	I	R		S	I	R	
Ciprofloxacin	14		91	86.7	8		92	92
Erythromycin	97		8	7.6	91	3	6	6
Tetracycline	22	2	81	77.1	39		61	61
Nalidixic acid	13		92	87.6	9	2	89	89
Gentamicin	105			0	100			0
Streptomycin	92	1	12	11.4	93		7	7

^1^ The total number of *Campylobacter* isolates from caecal samples tested for antimicrobial resistance was 105 and from neck skin samples was 100. ^2^ Number of susceptible (S), intermediate (I), and resistant (R) *Campylobacter* isolates identified by the disk diffusion method.

**Table 3:** Antimicrobial susceptibility patterns of *Campylobacter* isolates, identified by the disk diffusion method, according to the species ^1^.

Antimicrobial Agent	*Campylobacter Jejuni*				*Campylobacter Coli*			
	No. of *Campylobacter* Isolates ^2^			% of Resistant Isolates	No. of *Campylobacter* Isolates ^2^			% of Resistant Isolates
	S	I	R		S	I	R	
Ciprofloxacin	7		95	93.1	15		88	85.4
Erythromycin	94		8	7.8	94	3	6	5.8
Tetracycline	29	1	72	70.6	32	1	70	68
Nalidixic acid	7	1	94	92.2	15	1	87	84.5
Gentamicin	102			0	103			0
Streptomycin	90	1	11	10.8	94	1	8	7.7

^1^ The total number of *Campylobacter jejuni* was 102 and *Campylobacter coli* was 103. ^2^ Number of susceptible (S), intermediate (I), and resistant (R) *Campylobacter* isolates identified by the disk diffusion method.

## Data Availability

The data presented in this study are available on reasonable request from the corresponding author.
